# Supplementary material for: Complexome profiling of the Chlamydomonas psb28 mutant reveals TEF5 as an early PSII assembly factor
Source: Plant Cell. 2025 Mar 17;37(6):koaf055. doi: 10.1093/plcell/koaf055 (PMC12164590; doi:10.1093/plcell/koaf055)
Supplement: koaf055_Supplementary_Data [file koaf055_supplementary_data.zip › psb28_interactive.zip!/plots/intensity/qualityPlots/peptides/atpB_Cre-1.g2717034.t1.p1.html]

### Cre-1.g2717034.t1.p1

Trivial name: atpB  
SeqCoverage [median]: 76.80  
Mass: 53.16 kDa  
  
 

| Sequence | MissedCleavage | Mass [Da] | wtIntensity | muIntensity | Score |
| --- | --- | --- | --- | --- | --- |
| AHGGVSVFAGVGER | 0 | 1341.68 | 1521005.89 | 1279473.47 | 151.66 |
| AVSMNPTEGLMR | 0 | 1304.62 | 1433683.26 | 949832.75 | 253.88 |
| AVSMNPTEGLMRGMEVVDTGKPLSVPVGK | 2 | 2998.53 | 0.00 | 37162.19 | 86.30 |
| DGSITSIQAVYVPADDLTDPAPATTFAHLDATTVLSR | 0 | 3828.91 | 25115.59 | 0.00 | 81.24 |
| DVNKQDVLFFIDNIFR | 1 | 1982.03 | 355023.43 | 75691.59 | 197.29 |
| EGNDLYTEMK | 0 | 1198.52 | 205466.17 | 125320.33 | 237.92 |
| EGNDLYTEMKESGVIVEK | 1 | 2039.97 | 391265.89 | 267993.09 | 164.59 |
| ELQDIIAILGLDELSEEDRLIVAR | 1 | 2722.48 | 722.20 | 0.00 | 85.74 |
| ESGVIVEK | 0 | 859.47 | 929977.58 | 421910.85 | 176.09 |
| ESGVIVEKNLSDSK | 1 | 1503.78 | 2854.13 | 777.52 | 47.62 |
| FVQAGAEVSALLGR | 0 | 1416.77 | 850393.20 | 945174.34 | 237.67 |
| GIYPAVDPLESTSTMLQPWILGEK | 0 | 2644.35 | 165423.21 | 0.00 | 139.26 |
| GMEVVDTGK | 0 | 934.44 | 1452.89 | 688.92 | 78.52 |
| GMEVVDTGKPLSVPVGK | 1 | 1711.92 | 2786563.76 | 2220875.82 | 158.45 |
| GMEVVDTGKPLSVPVGKVTLGR | 2 | 2238.24 | 1949.14 | 3294.41 | 72.64 |
| GQVPNIYNALTIR | 0 | 1457.80 | 1181151.25 | 1217266.88 | 248.21 |
| GQVPNIYNALTIRAK | 1 | 1656.93 | 0.00 | 4608.43 | 47.20 |
| HYDSAQSVK | 0 | 1033.48 | 571807.55 | 427699.51 | 241.96 |
| HYDSAQSVKK | 1 | 1161.58 | 810579.67 | 610239.75 | 216.26 |
| IFNVLGEPVDNMGNVK | 0 | 1744.88 | 873418.85 | 879620.82 | 244.71 |
| IFNVLGEPVDNMGNVKVEETLPIHR | 1 | 2819.46 | 247234.87 | 392459.38 | 152.07 |
| IGLFGGAGVGKTVLIMELINNIAK | 1 | 2427.39 | 3203.72 | 1527.05 | 107.47 |
| IVQIIGPVLDIVFAK | 0 | 1624.00 | 422649.97 | 140648.38 | 161.25 |
| LSIFETGIK | 0 | 1006.57 | 2283880.61 | 3221335.14 | 225.24 |
| LSIFETGIKVVDLLAPYRR | 2 | 2189.26 | 1895.54 | 10515.42 | 73.28 |
| MPSAVGYQPTLATEMGGLQER | 0 | 2235.07 | 1058304.76 | 1487744.65 | 233.38 |
| MRVALTALTMAEYFR | 1 | 1771.91 | 40066.45 | 104622.60 | 150.59 |
| MRVALTALTMAEYFRDVNK | 2 | 2228.14 | 0.00 | 6408.37 | 78.23 |
| NLSDSKVALVYGQMNEPPGAR | 1 | 2245.12 | 8125.49 | 27516.56 | 171.83 |
| PLSVPVGK | 0 | 795.49 | 0.00 | 1266.43 | 68.92 |
| QDVLFFIDNIFR | 0 | 1525.79 | 21473.00 | 6237.69 | 237.84 |
| TAPAFVDLDTR | 0 | 1204.61 | 2333657.82 | 3481410.06 | 241.61 |
| TREGNDLYTEMK | 1 | 1455.67 | 281959.75 | 187553.78 | 174.72 |
| TREGNDLYTEMKESGVIVEK | 2 | 2297.12 | 478555.48 | 431678.06 | 196.36 |
| TVLIMELINNIAK | 0 | 1470.85 | 1251050.48 | 1198963.72 | 347.46 |
| VALTALTMAEYFR | 0 | 1484.77 | 1296210.11 | 1107819.22 | 299.28 |
| VALTALTMAEYFRDVNK | 1 | 1941.00 | 15847.99 | 28681.00 | 120.30 |
| VALVYGQMNEPPGAR | 0 | 1600.80 | 1100672.14 | 721555.56 | 314.08 |
| VEETLPIHR | 0 | 1092.59 | 896935.89 | 823534.44 | 171.36 |
| VVDLLAPYR | 0 | 1044.60 | 1266951.85 | 1380445.91 | 187.80 |
| VVDLLAPYRR | 1 | 1200.70 | 1494064.65 | 1635865.97 | 171.36 |
| YVSLAETIEGFGK | 0 | 1412.72 | 770.52 | 0.00 | 85.38 |
|  |  |  | 26611364.8 | 25865420.1 |  |
